# Acceleration of sarcopenia in elderly patients who develop asymptomatic pneumonia shadow within one year after surgery for early gastric cancer

**DOI:** 10.1186/s12893-023-02096-2

**Published:** 2023-08-11

**Authors:** Ayako Kamiya, Tsutomu Hayashi, Ryota Sakon, Kenichi Ishizu, Takeyuki Wada, Sho Otsuki, Yukinori Yamagata, Hitoshi Katai, Takaki Yoshikawa

**Affiliations:** Department of Gastric Surgery, National Cancer Center Hospital, 5-1-1 Tsukiji, Chuo-ku, Tokyo, 104-0045 Japan

**Keywords:** Elderly, Gastrectomy, Pneumonia, Sarcopenia, Skeletal muscle

## Abstract

**Background:**

Although early gastric cancer is curable with local treatment, the overall survival in elderly patients did not reach 80% at five years after surgery. The major cause of death in elderly patients with early gastric cancer is not cancer itself but is related to postoperative sarcopenia. Elderly patients frequently develop postoperative asymptomatic pneumonia shadow, which is associated with a poor prognosis. However, why asymptomatic pneumonia shadow worsens the prognosis remains unclear. We investigated whether sarcopenia is accelerated in patients who developed asymptomatic pneumonia shadow.

**Methods:**

We retrospectively examined patients of > 75 years of age who underwent R0 gastrectomy for gastric cancer and were diagnosed with T1 disease at National Cancer Center Hospital between 2005 and 2012. The diagnosis of asymptomatic pneumonia shadow was defined by diagnostic findings of pneumonia (consolidation type, reticular type, and nodular type) which were newly observed on chest computed tomography performed one year after surgery in comparison to preoperative computed tomography. Postoperative muscle loss was assessed by a computed tomography-based analysis using the L3 skeletal muscle index before and two years after surgery and the rate of decrease was calculated. Patients were classified into two groups according to the rate of decrease (cut-off value: 10%).

**Results:**

Of the 3412 patients who underwent gastrectomy in our hospital during the study period, 142 were included in this study. Asymptomatic pneumonia shadow was found in 26 patients (18%). Patients who developed asymptomatic pneumonia shadow showed a significantly greater loss of muscle volume in comparison to patients who did not develop asymptomatic pneumonia shadow. In the multivariate analysis, total gastrectomy and asymptomatic pneumonia shadow were the independent risk factors for severe muscle loss. However, there was no significant difference in prognosis between the two groups.

**Conclusions:**

Sarcopenia was accelerated in elderly patients who developed asymptomatic pneumonia shadow after surgery for early gastric cancer. However, the poor prognosis in these patients may not be related to accelerated sarcopenia.

## Background

In Japan, gastric cancer is characterized by early stage at the diagnosis and elderly age [1, 2]. Although early gastric cancer is curable with local treatment, the overall survival in elderly patients did not reach 80% at five years after surgery [3]. It would be explained by the high proportion of deaths from causes other than gastric cancer itself [4].

Sarcopenia has been defined as the loss of muscle mass and strength that occurs with aging [5]. Sarcopenia is associated with increased adverse outcomes including falls, functional decline, frailty, and mortality [6]. The prevalence of sarcopenia was reported to be approximately 22% of men and women aged 75–79 years old and 32.4% of men and 47.7% of women aged 80 years and older [7]. Loss of body weight is a common and causes serious outcome in patients with gastric cancer who have undergone gastrectomy. Takahashi et al. reported that 6% of the elderly patients were diagnosed with sarcopenia preoperatively and it increased to 22% by 1 year after gastrectomy [8]. Sarcopenia is gradually being recognized as a resistance factor in cancer treatment. Some studies have shown that skeletal muscle loss after gastrectomy could predict a poor prognosis in gastric cancer patients [9–11].

In the elderly population, pneumonia is one of the most frequent causes of death [12]. We previously investigated asymptomatic pneumonia shadow (APS) on chest computed tomography (CT) at regular follow-up in elderly patients who received surgical treatment for early gastric cancer [13, 14]. Surprisingly, this pneumonia shadow was detected in approximately one-quarter of the elderly patients [13] and APS was significantly associated with poor survival [14]. However, it remains unclear why APS worsens the prognosis despite being a silent shadow without active symptoms. If APS accelerates sarcopenia, it could worsen the prognosis.

To clarify whether sarcopenia is accelerated in patients who develop APS, we compared the change of muscle volume in the 2 years after gastrectomy between elderly patients who developed APS at 1 year after surgery and those who did not. Moreover, we also clarified whether APS is a significant risk factor for sarcopenia in the 2 years after surgery.

**Fig. 1:**
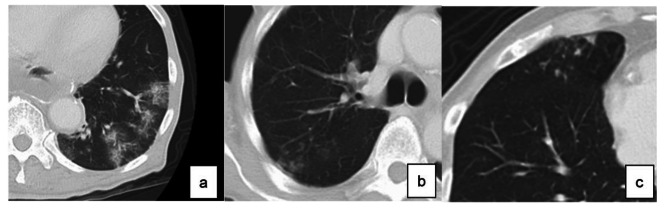
Definition of the diagnosis of pneumonia based on CT consolidation type, b. reticular type, c. nodular type

## Methods

### Patients

Patients were selected from a clinical database of consecutive patients who received gastrectomy for gastric cancer at the National Cancer Center Hospital between January 2005 and December 2012, and who met the following criteria: (1) a pathological diagnosis of T1, (2) age ≥ 75 years, (3) R0 resection achieved, and (4) chest to abdominal CT performed before surgery and within two years after surgery.

**Fig. 2:**
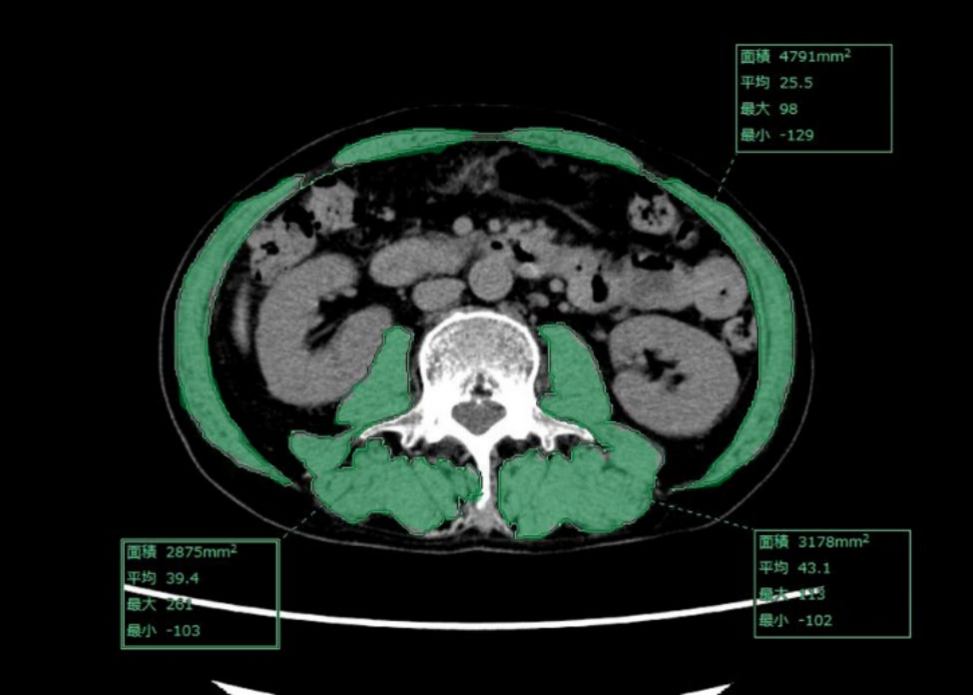
Axial computed tomography slice of the third lumbar vertebra (L3). Green areas indicate skeletal muscle

### Surgery and follow-up

The treatment strategy for early-stage gastric cancer was determined by the Japanese Gastric Cancer Treatment Guideline version 2 or version 3 depending on the date of the surgery [15, 16]. In summary, gastrectomy with D1 or D1 + lymphadenectomy was performed according to the tumor characteristics, without adjuvant chemotherapy, regardless of the patient’s age. Postoperative follow-up evaluations, including physical examinations, blood tests, and CT or ultrasound, were basically performed every six months for the first year and then every year for the next four years. As routine oncologic follow-up, CT of the chest to the abdomen was basically performed every year for at least five years postoperatively. Additional imaging studies were performed if recurrence was suspected.

**Fig. 3:**
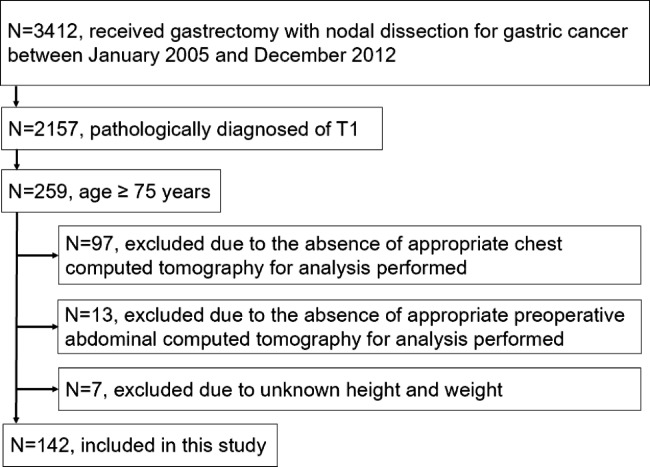
Flow diagram of the present study

### Diagnostic criteria for asymptomatic pneumonia shadow

APS was diagnosed on follow-up CT in the first year after surgery. According to the guidelines for diagnostic imaging of adult community-acquired pneumonia 2007, diagnostic findings of pneumonia on CT were classified into three types: consolidation type, reticular type, and nodular type, and those presenting with two or more types at the same time were called mixed type (Fig. 1) [17]. The diagnosis of APS was defined by these findings which were newly observed on chest CT performed 1 year after surgery in comparison to preoperative CT. After certified radiologists confirmed the radiological findings, two surgeons evaluated the images to determine the presence or absence of APS. If the two surgeons’ diagnoses differed, the images were evaluated again to make a final determination.

**Fig. 4:**
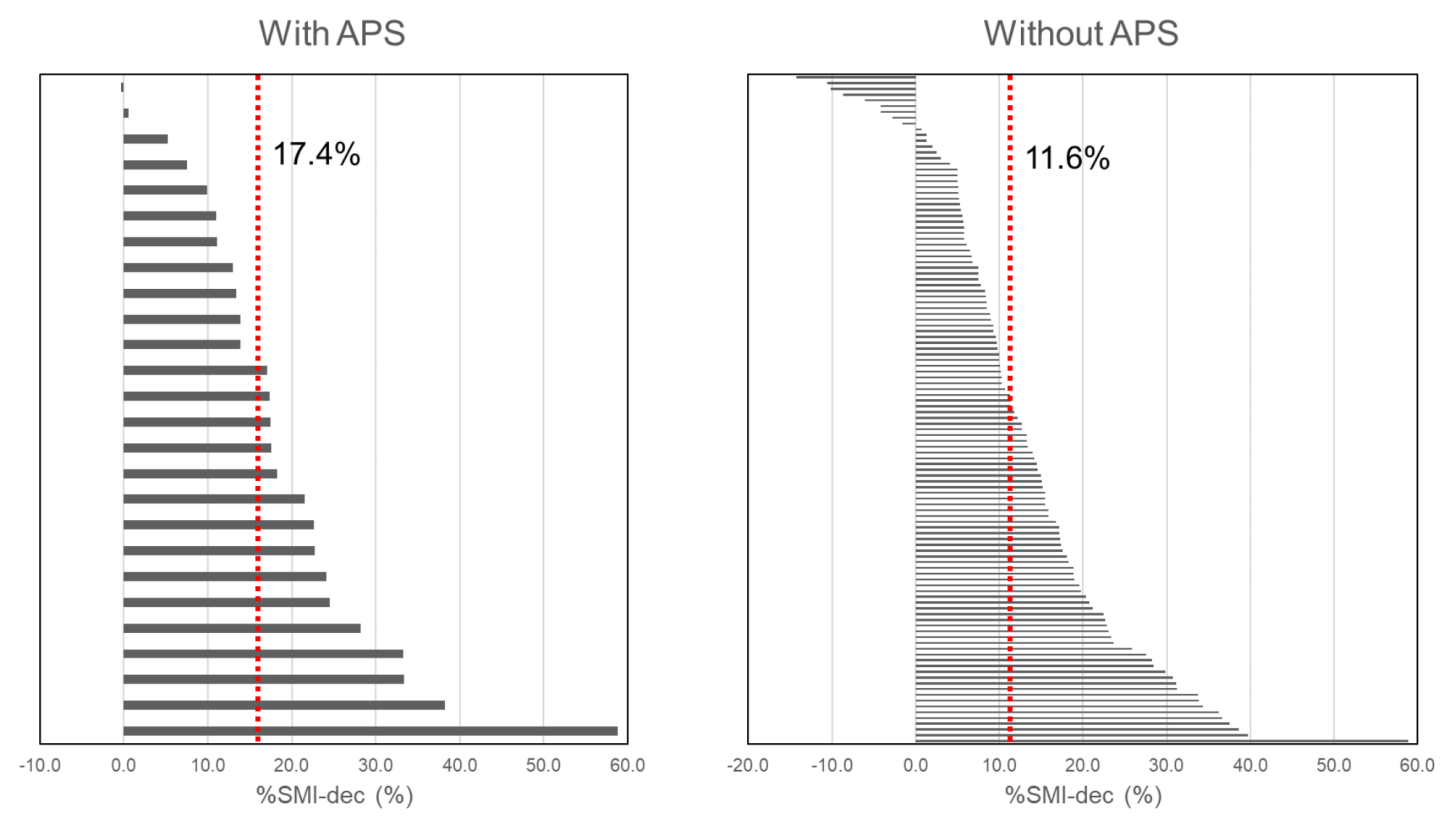
%SMI-dec of the patients with and without APS

### Evaluation of decreased muscle mass

We performed a CT-based analysis and used the muscle area measured on CT [18]; axial slices of the third lumbar vertebrae (L3) were evaluated [19]. The sum of the cross-sectional areas of the skeletal muscles including the psoas, paraspinal, and abdominal wall muscles was calculated using the SYNAPSE VINCENT system (Fig. 2). The skeletal muscle area in a single abdominal image is proportional to the whole-body muscle mass [20]. The muscle area normalized by the square of the height [m] is called the L3 skeletal muscle index (SMI, [cm^2^/m^2^]) [21]. We investigated the L3 SMI before surgery and at 2 years after surgery and calculated the rate of decrease in the L3 SMI (%SMI-dec) as follows: %SMI-dec = 100 x (SMI before surgery - SMI 2 years after surgery) / SMI before surgery. Considering the clinical significance of %SMI-dec, we set the cut-off value as 10%. Then, the patients were classified into those with high %SMI-dec (H group) and those with low %SMI-dec (L group).

**Fig. 5:**
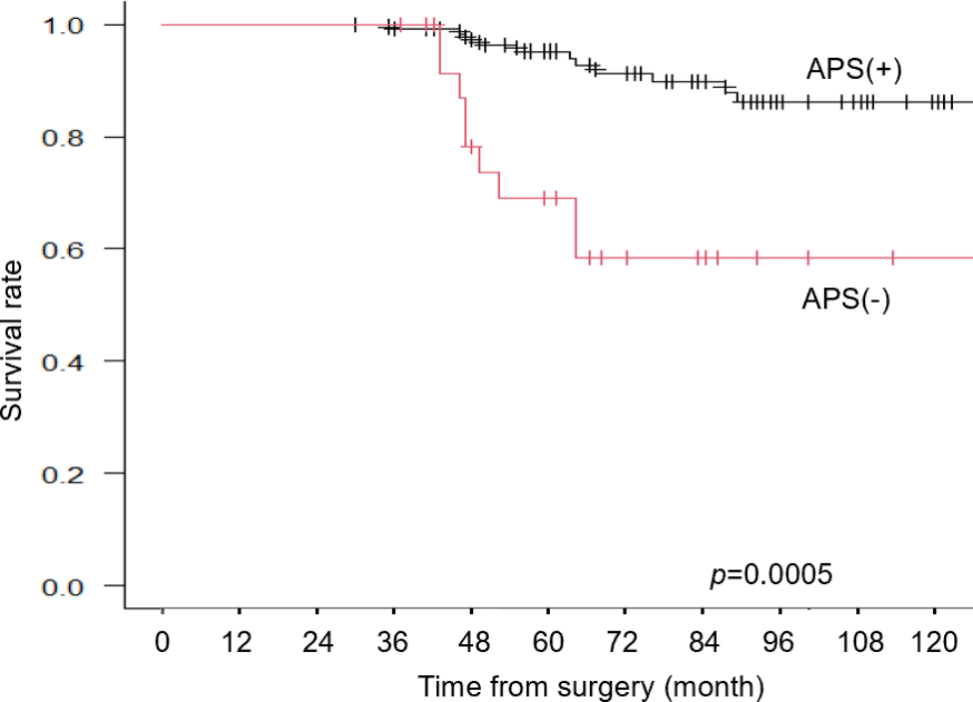
Over-all survival curve of the patients with APS and those without APS

**Fig. 6:**
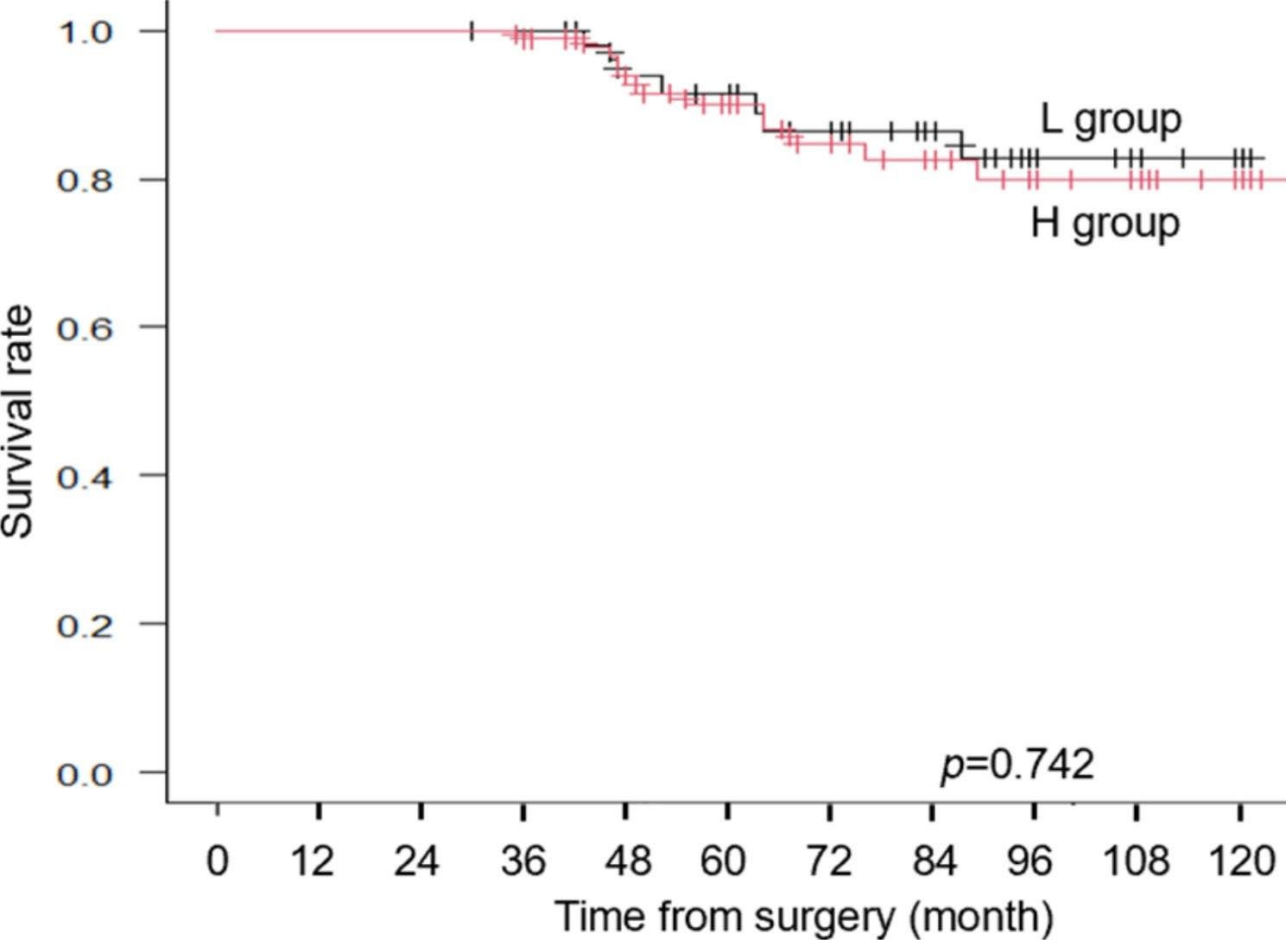
Over-all survival curve of the patients with high %SMI-dec (H group) and those with low %SMI-dec (L group)

### Statistical analysis

All statistical analyses were performed using the SPSS software program (Statistical Package for the Social Sciences version 15.0; SPSS, Chicago, IL, USA). Statistical comparisons of the differences in the age, body mass index (BMI = body weight [kg]/height [m]^2^), Geriatric Nutritional Risk Index (GNRI = 4.89×Alb [g/dl] + 41.7×(body weight [kg] / ideal body weight [kg]), vital capacity (%VC) and forced expiratory volume in 1 s (FEV1%) were analyzed by Student’s t-test, and other variables including the Charlson score [22] were analyzed by the chi-squared test.

To identify risk factors for high %SMI-dec, age, surgical procedure, Charlson score, BMI and GNRI were converted to binary data. The median GNRI was used as the cut-off value. Statistical comparisons of differences in each variable between the L and the H groups were performed using the chi-squared test. Variables were also investigated by a multivariate logistic regression analysis to assess the risk factors associated with high %SMI-dec. *P* values of < 0.05 were considered to indicate statistical significance.

The overall survival (OS) curves were calculated based on the Kaplan–Meier curves and were compared by the log-rank test. The data for patients who did not experience an event by the date of the final observation were treated as censored cases.

## Results

### Patients’ demographics

Among the 3412 patients who underwent gastrectomy for gastric cancer at National Cancer Center Hospital, we reviewed the records of 142 patients who had adequate CT images taken before and after surgery. No patients had treated by neoadjuvant chemotherapy or postoperative adjuvant chemotherapy. Figure 3 shows a consort diagram of the present study. APS was found in 26 patients (18%) by chest CT: consolidation type (n = 11), reticular type (n = 3), nodular type (n = 10), and mixed type (n = 2).

Table 1 summarizes the clinicopathological characteristics of the patients. All patients who underwent distal gastrectomy were reconstructed with Roux-en-Y, and all patients who underwent proximal gastrectomy were reconstructed with jejunal interposition. In comparison to patients without APS, the patients with APS included a high proportion of male and a high proportion of patients with preoperative pneumonia shadow. The other background characteristics of the groups were similar.

**Table 1:** Clinicopathological characteristics

Variables	Total N (%)^*^	APS^†^ (-) (n = 116) N (%)^*^	APS^†^ (+) (n = 26) N (%)^*^	p value^**^
Age (Median, range)	77 (75–87)	77 (75–87)	77 (75–83)	0.808
Sex				0.123
Male	90 (63)	70 (60)	20 (77)	
Female	52 (37)	46 (40)	6 (23)	
Surgical approach				0.695
Open	130 (92)	105 (91)	25 (96)	
Laparoscopy	12 (8)	11 (9)	1 (4)	
Surgical procedure				0.861
Distal gastrectomy	65 (46)	53 (46)	12 (46)	
Pylorus preserving gastrectomy	43 (30)	35 (30)	8 (30)	
Proximal gastrectomy	12 (8)	9 (8)	3 (12)	
Total gastrectomy	22 (16)	19 (16)	3 (12)	
BMI (Median, range) ^††^	22.4 (16.5–32.2)	22.5 (16.7–32.2)	22.1 (16.5–30.5)	0.652
GNRI (Median, range) ^†††^	104.5 (89.0-128.0)	104.5 (89.0-125.3)	104.0(89.9–128.0)	0.791
Charlson Score				0.726
0	82 (58)	69 (59)	13 (50)	
1	27 (19)	22 (19)	5 (20)	
2	20 (14)	15 (13)	5 (20)	
3	7 (5)	5 (4)	2 (7)	
4	2 (1)	2 (2)	0 (0)	
5	0 (0)	0 (0)	0 (0)	
6	3 (2)	2 (2)	1 (3)	
7	1 (1)	1 (1)	0 (0)	
Current smoking history				0.695
+	57 (40)	48 (41)	9 (35)	
-	85 (60)	68 (59)	17 (65)	
%VC (Median, range) ^††††^	104 (53–175)	104 (53–175)	103 (75–132)	0.99
FEV1% (Median, range)^†††††^	73.5 (34–96)	73.5 (34–92)	74 (58–96)	0.386
Sliding hernia				1.000
+	44 (31)	36 (31)	8 (31)	
-	98 (69)	80 (69)	18 (69)	
Preoperative pneumonia				0.129
+	62 (44)	47 (41)	15 (58)	
-	80 (56)	69 (59)	11 (42)	
Pathological TNM stage				0.346
T1aN0	59(41)	49 (42)	10 (38)	
T1aN1	1 (1)	0(0)	1(4)	
T1bN0	71 (50)	58 (50)	13 (50)	
T1bN1	11 (8)	9 (8)	2 (8)	

*: Number and percentage of patients in each category in each group (except age, BMI, GNRI, %VC and FEV1%)

^**^: Age, BMI, GNRI, %VC and FEV1% were analyzed by Student’s t-test, and other variables were analyzed by the chi-squared test

APS^†^: asymptomatic pneumonia shadow

BMI^††^: Body Mass Index= (body weight (kg)/ height (m^2^))

GNRI^†††^: Geriatric Nutritional Risk Index = 14.89×Alb (g/dl) + 41.7×(body weight (kg)/ ideal body weight (kg))

%VC^††††^: Vital capacity

FEV1%^†††††^: Forced expiratory volume in 1 s

Figure 4 shows the %SMI-dec of the patients with and without APS. The median %SMI-dec in patients with and without APS was 17.4 and 11.6, respectively (*p* = 0.035).

### Risk factors for high %SMI-dec

To identify risk factors for high %SMI-dec, variables were converted to binary data (Table 2). Eighty-nine patients (63%) were included in the H group. The surgical procedures were classified into two groups by the presence or absence of the residual stomach (distal gastrectomy, pylorus preservastrectomy and proximal gastrectomy). In our study population, 15% of patients received total gastrectomy. Among the variables included in the univariate analyses, APS was the only factor that showed a significant difference between the L and H groups (p = 0.043). In the multivariate analysis, the surgical procedure and APS were independent risk factors for high %SMI-dec (p = 0.025 and p = 0.043, respectively).

**Table 2:** Risk factors for high %SMI^†^-dec^††^ according to univariate and multivariate analyses

Variables	Total N (%)^*^	L group N (%)^*^	H group N (%)^*^	Univariate analysis	Multivariate analysis		
				*p* ^**^	Odds ratio	*p* ^***^	95% confidence interval
Age				0.187	1.850	0.136	0.824–4.170
<80	100 (70)	41 (77)	59 (66)				
80≤	42 (30)	12 (23)	30 (34)				
Sex				0.593			
Male	90 (63)	32 (60)	58 (65)				
Female	52 (37)	21 (40)	31 (35)				
Surgical procedure				0.055	3.802	0.025	1.181–12.20
DG^†††^+PPG^††††^+PG^†††††^	120 (85)	49 (92)	71 (80)				
TG^††††††^	22 (15)	4 (8)	18 (20)				
Charlson score				1.000			
<3	129 (91)	48 (91)	81 (91)				
3≤	13 (9)	5 (9)	8 (9)				
BMI^†††††††^				0.488			
<22.5	71 (50)	29 (55)	42(47)				
22.5≤	71 (50)	24 (45)	47(53)				
GNRI^††††††††^				0.862			
<104.5	67 (47)	26 (49)	41 (46)				
104.5≤	75 (53)	27 (51)	48 (54)				
APS^†††††††††^				0.043	2.990	0.043	1.030–8.660
+	26 (18)	5 (9)	21 (24)				
-	116 (82)	48 (91)	68 (76)				

^*^: The number and percentage of patients in each category in each group

^**^: Variables were analyzed by a chi-squared test

^***^: Variables were analyzed by a logistic regression analysis

SMI^†^: skeletal muscle index = L3 skeletal muscle area (cm^2^)/ height (m)^2^

%SMI-dec^††^: rate of decrease in L3 SMI = 100 x (SMI before surgery - SMI 2 years after surgery) / SMI before surgery

DG^†††^: distal gastrectomy

PPG^††††^: pylorus preserving gastrectomy

PG^†††††^: proximal gastrectomy

TG^††††††^: total gastrectomy

BMI^†††††††^: Body Mass Index = body weight (kg)/ height (m)^2^

GNRI^††††††††^: Geriatric Nutritional Risk Index = 14.89×Alb (g/dl) + 41.7×(body weight (kg)/ ideal body weight (kg))

APS^†††††††††^: asymptomatic pneumonia shadow

### Survival outcomes

The median follow-up period from the date of surgery was 77 months (range, 30–132 months). In the Kaplan-Meier survival analysis, patients with APS experienced significantly shorter OS (p = 0.0005) than those without APS (Fig. 5). However, comparing H group and L group there was no significant difference in the survival between the two groups. (p = 0.742, Fig. 6).

## Discussion

Among elderly patients with Stage I gastric cancer, deaths due to other diseases were frequently observed in the long period after surgery [4]. The prevalence of sarcopenia was reported to be approximately 22% of men and women aged 75–79 years old and 32.4% of men and 47.7% of women aged 80 years and older [7].

In the present study, we examined the relationships between postoperative muscle loss and APS in elderly patients after gastrectomy for early gastric cancer. We found that muscle loss in the 2 years after surgery was significantly greater in patients who developed APS in comparison to those who did not. Moreover, APS and total gastrectomy were independent risk factors related with muscle loss exceeding 10% in the 2 years after gastrectomy. The patients who had accelerated sarcopenia 2 years after surgery had not so poor prognosis as compared with those who had not.

Patients who developed APS showed a significant loss of muscle volume in the 2 years after surgery in comparison to those who did not. Moreover, APS was a significant risk factor related with postoperative muscle loss. Why was sarcopenia accelerated in patients having APS despite APS being a silent shadow without active symptoms? APS might reflect the presence of mild pneumonia. Even mild inflammation may result in a loss of muscle mass. Pneumonia has been reported to induce muscle atrophy in an animal model and in the elderly human population [23–25]. The skeletal muscle loss induced by pneumonia might cause a vicious cycle with the repeated episodes of pneumonia and further muscle atrophy.

In this study, we could confirm that patients who developed APS had the poor prognosis as compared with those who did not as described in the previous study [14]. However, the patients who have accelerated sarcopenia had not so poor prognosis as compared with those who had not. So, accelerated sarcopenia was not directly related to poor prognosis, which is contradictory to the previous studies [9–11]. This difference could be explained by the difference of the cohort. In the previous reports, the cohort was not limited to the elderly patients. In the elderly patients, sarcopenia is frequently observed. When the sarcopenia was defined as the cut-off values at 49.2 cm^2^ /m^2^ for males and 35.7 cm^2^ /m^2^for females, respectively, as described in the previous study [13], 69% of the patients in this cohort had sarcopenia before surgery. Moreover, 89% of the patients had sarcopenia 2 years after surgery. Thus, even the elderly patients who did not have accelerated sarcopenia had been in sarcopenia 2 years after surgery. Degree of sarcopenia may not be related with prognosis.

In addition to APS, total gastrectomy was also a significant risk factor for muscle depletion in the 2 years after surgery. Previously, several investigators also showed that total gastrectomy was a risk factor for postoperative muscle loss [9, 10, 26, 27]. Kim et al. reported that female sex, preoperative weight loss, proximal location of the tumor and differentiated tumor were significant risk factors for post-gastrectomy sarcopenia [9]. Yamazaki et al. also demonstrated that total gastrectomy was independently associated with severe muscle loss [26]. The severe muscle loss after total gastrectomy might be attributed to reduced oral intake. Two reasons for this reduced oral intake could be considered, namely loss of retention ability and/or decreased ghrelin (an appetizing hormone that is released from the stomach) [28].

The present study was associated with some limitations. First, this was a retrospective study based on data from a single institution that analyzed 142 elderly patients with early gastric cancer. Longitudinal larger data are required to confirm the present results. Second, we only examined muscle volume and did not measure muscle strength. Therefore, we could not evaluate whether APS reduced the muscle function. Third, this was a cross-sectional study, which thereby makes it difficult to clarify the temporal or causal relationship between skeletal muscle loss and APS.

## Conclusions

Sarcopenia was accelerated in elderly patients who developed APS after surgery for early gastric cancer. APS was a significant risk factor associated with severe muscle loss after surgery. However, the poor prognosis in these patients may not be related to accelerated sarcopenia.

## Data Availability

All data analyzed during this study are included in this published article.

## List of Abbreviations

APS: Asymptomatic pneumonia shadow
CT: Computed tomography
L3: The third lumbar vertebrae
SMI: Skeletal muscle index
%SMI-dec: The rate of decrease in the L3 SMI
BMI: Body mass index
GNRI: Geriatric Nutritional Risk Index
% VC: % Vital capacity
FEV1%: Forced expiratory volume % in 1 s
OS: Overall survival
